# The potential of computerised analysis of bowel sounds for diagnosis of gastrointestinal conditions: a systematic review

**DOI:** 10.1186/s13643-018-0789-3

**Published:** 2018-08-17

**Authors:** Andrisha-Jade Inderjeeth, K. Mary Webberley, Josephine Muir, Barry J. Marshall

**Affiliations:** 1 North Metropolitan Health Service, Sir Charles Gairdner Hospital, Nedlands, Western Australia Australia; 2 The Marshall Centre for Infectious Diseases Research and Training, School of Biomedical Sciences, QEII Medical Site, The University of Western Australia, Perth, Western Australia Australia

**Keywords:** Bowel sound, Borborygmi, Diagnostics, Systematic review, Gastrointestinal medicine, Non-invasive tests, Research quality assessment methodology post-operative ileus

## Abstract

**Background:**

Gastrointestinal (GI) conditions are highly prevalent, and their standard diagnostic tests are costly and carry risks. There is a need for new, cost-effective, non-invasive tests. Our main objective was to assess the potential for use of bowel sounds computerised analysis in the diagnosis of GI conditions.

**Methods:**

The systematic review followed the PRISMA requirements. Searches were made of four databases (PubMed, MEDLINE, Embase, and IEEE Xplore) and the references of included papers. Studies of all types were included. The titles and abstracts were screened by one author. Full articles were reviewed and data collected by two authors independently. A third reviewer decided on inclusion in the event of disagreement. Bias and applicability were assessed via a QUADAS tool adapted to accommodate studies of multiple types.

**Results:**

Two thousand eight hundred eighty-four studies were retrieved; however, only 14 studies were included. Most of these simply assessed associations between a bowel sound feature and a condition. Four studies also included assessments of diagnostic accuracy. We found many significant associations between a bowel sound feature and a GI condition. Receiver operating characteristic curve analyses revealed high sensitivity and specificity for an irritable bowel syndrome test, and a high negative predictive value for a test for post-operative ileus. Assessment of methodological quality identified weaknesses in all studies. We particularly noted a high risk of bias in patient selection. Because of the limited number of trials included and the variety in conditions, technology, and statistics, we were unable to conduct pooled analyses.

**Conclusions:**

Due to concerns over quality and small sample sizes, we cannot yet recommend an existing BSCA diagnostic test without additional studies. However, the preliminary results found in the included studies and the technological advances described in excluded studies indicate excellent future potential. Research combining sophistical clinical and engineering skills is likely to be fruitful.

**Systematic review registration:**

The review protocol (review ID number 42016054028) was developed by three authors (AI, KMW, and JM) and was published in the PROSPERO International prospective register of systematic reviews. It can be accessed from https://www.crd.york.ac.uk/PROSPERO/.

**Electronic supplementary material:**

The online version of this article (10.1186/s13643-018-0789-3) contains supplementary material, which is available to authorized users.

## Background

Gastrointestinal (GI) disease and disorders are significant causes of morbidity worldwide. For example, inflammatory bowel diseases lead to around 100,000 hospital admissions annually in the USA [1], whilst irritable bowel syndrome (IBS) is the second most common cause of work absenteeism [2] and accounts for up to 50% of gastroenterology outpatient clinic time [3].

Accurate diagnosis of GI pathology typically requires a gastroenterology review (prolonging waiting lists) prior to invasive procedures such as endoscopies, biopsies, and manometry. Indeed, the gold standard for positive diagnosis of chronic GI diseases is often endoscopy with biopsy of tissue for analysis. Unfortunately, patients with functional gastrointestinal disorders such as IBS typically also endure invasive endoscopies to diagnose these conditions by means of exclusion of more sinister pathologies [4].

However, endoscopies carry a small risk of gastrointestinal perforation—requiring emergency surgery and carrying high mortality rates [5]. Other risks associated with endoscopy such as bleeding, infection, and anaesthetic complications, whilst rare, can be life threatening. In addition to these risks, the costs to the patient include physical discomfort, psychological distress, and time off-work.

The cost of unnecessary endoscopies to health care systems also cannot be underestimated; associated theatre time and adequate nursing and medical staffing are all expensive. By avoiding unnecessary ‘normal’ endoscopies, reallocation of staffing, resources, and funding would allow better provision of urgent care to the patients with life-threatening conditions such as malignancy or GI bleeding.

Similarly, certain GI conditions such as bowel obstructions or post-operative ileus are often investigated with abdominal imaging involving radiation exposure with associated risks.

Clearly, there is a significant need for cost-effective, non-invasive diagnostic tests for GI diseases that avoid unnecessary patient risk and place less strain on the health care system.

Auscultation of bowel sounds is a traditional technique pioneered by Cannon in the early twentieth century [6] and widely taught to training doctors, despite limited clinical value and relevance due to inaccuracy and variability in interpretation [7–9]*.*

Technological advancement in the twenty-first century has brought a new era to the practice of medicine, minimising human error and variation in the interpretation of data. Increases in computer processing power and improvement in data analysis bring the potential for practice-altering research into bowel sounds analysis for evaluating GI motility. Can the new technology outperform clinicians in analysing the myriad of sounds emanating from the GI tract?The first objective of this study was to evaluate if bowel sound computational analysis (BSCA) is currently useful as a tool in GI condition diagnosis. Table 1 provides the study inclusion criteria in terms of population, index test, reference test, and diagnosis (PIRD) for this review question.

**Table 1:** Eligibility criteria: general inclusion criteria and those shaped by the PIRD and PICO components

All studies	Inclusion criteria		
Publication details	English, abstract, original study, peer-reviewed article, or conference proceeding, any year. Human subjects any age, but not foetal. Any setting.		
DTA studies	Inclusion criteria	Preliminary (non-DTA) studies	Inclusion criteria
Population	Patients with a diagnosis or subjective symptoms of a GI condition (pathological or functional).	Population	Patients with a diagnosis or subjective symptoms of a GI condition (pathological or ‘functional’) and healthy controls. Some studies had groups for multiple conditions.
Index test	Computerised analysis of bowel sounds	Index test	Computerised analysis of bowel sounds
Reference test	Standard method used for diagnosis	Comparator(s)	Healthy controls or different target condition groups diagnosed by standard methods
Diagnosis	Confirmed or excluded diagnosis of GI condition(s)	Outcome	Results of statistical analysis testing for heterogeneity or associations between bowel sound feature and condition(s)

Diagnostic test accuracy (DTA) studies were defined as those studies providing some measure of accuracy for the index test, such as sensitivity, specificity, negative predictive value, positive predictive value, accuracy, positive and negative likelihood ratios, or ROC analysis. The preliminary studies were typically proof-of-concept studies aimed at assessing an association between a bowel sound feature and a GI condition, rather than assessing accuracy in a clinical setting

Given the limited publications, heterogeneity of studies, and paucity of proper diagnostic test accuracy (DTA) studies, our secondary objective was to assess if BSCA is likely to be useful in the future given the rate of technological advancement. Hence, we also assessed if bowel sound computational analysis revealed signature patterns or variation associated with gastrointestinal conditions. Again Table 1 provides included study characteristics in terms of population, index test, comparator, and outcome (PICO terms). We hypothesise that, using modern techniques of noise-removal and sound analysis, computerised analysis of bowel sounds has the potential to be a non-invasive technique to aid in the screening and diagnosis of gastrointestinal conditions.

## Methods

### Aim

The aim of this review was to assess the potential for use of bowel sounds computerised analysis in the diagnosis of GI conditions. We looked at the value of specific tests in the clinical setting, as well as the potential of the general approach. Reporting followed PRISMA guidelines. The completed checklist is provided as Additional file 1.

### Eligibility criteria

We included studies of any type and in any setting where BSCA was used as a tool for identification or characterisation of GI conditions (clinical trials of devices; diagnostic test accuracy studies, both single gate or case-control; studies with retrospective diagnosis from the data; and preliminary observational studies with tests for associations between bowel sound characteristics and GI conditions or heterogeneity across groups). This reflects the full breadth of studies found along the development pathway of a diagnostic test from proof-of-concept through to widespread clinical use. We included original studies (not reviews) on human subjects, published in a peer-reviewed journal in English. The publication had to have an abstract, to allow the first stage of our search protocol and as an indicator of the depth of the study. We included conference proceedings from the search of engineering journals, given these are typically both comprehensive and peer-reviewed. The participants could be any age, excluding studies with foetal subjects.

Other inclusion criteria were shaped by the PIRD terms for the DTA studies (which we defined as any study that produced accuracy measures, such as sensitivity, specificity) and PICOS terms for the simpler preliminary studies (see Table 1). Our analysis included no limitations on year of publication and inclusion criteria were broad, so as to identify a comprehensive list of relevant studies.

### Information sources

Searches were made of electronic databases with the last search made on 7 April 2017. We also searched for additional studies by reading the references of each included paper.

#### Electronic searches

After a preliminary review of PubMed for existing published terminology, our search strategy was developed in consultation with an Information Specialist from the University of Western Australia, as well as several team members on the project including both clinicians and engineers. Searches were made of four major databases: PubMed, MEDLINE, IEEE Xplore, and Embase. Search terms broadly related to the three key features required by studies to address the review aims: anatomical site in question (e.g. bowel), measure of sound (e.g. auscultation/telemetry), and technology used for analysis (e.g. computational analysis).

The electronic search strategy for Embase, PubMed, and Medline was:

((bowel or stomach or gut or gastrointestinal or abdominal or intestinal or intestine or bowel-sound or bowels) and (sounds or sound or noise or noises or borborygmus or borborygmi or bowel-sound or bowel-sounds) and (telemetry or biosensor or acoustic or microphone or analysis or enterotachogram or pattern analysis or wavelets or wavelet or motility analysis or neural networks or neural network or computerised auscultation or electronic stethoscope or computer analysis or computerized analysis or computerised analysis or computerized phonoenterography or computerized phonoenterography or computerised auscultation or computerized auscultation or enterotachogram or wavelet-based or monitoring or pattern analysis or auscultation or phonogram)). Limits for Embase were human, English, and journal articles. Limits for MEDLINE were human, English, journal articles, and abstracts, and for PubMed, limits were English and human and limits.

IEEE allows less search terms than the previous databases; hence, the following terms were used:

bowel sound* or abdom* vibration* and signal processing.

#### Selection process

All articles were initially screened by one reviewer (AI) and excluded based on title and then screened and excluded based on abstract (AI). In the event of uncertainty of inclusion at either of these points, a second reviewer (KMW) aided with review of both title and abstract and a consensus decision. Two independent reviewers assessed the remaining full articles for review eligibility with regard to pre-determined inclusion and exclusion criteria as outlined in Table 1 (AI and KMW). Articles were included if a consensus decision was reached by both reviewers (AI and KMW) with study data inputted onto independent spreadsheets prior to quality assessments. In the event of disagreement, the decision was made by a third reviewer (BJM), after discussion.

### Data collection process and data items

Two reviewers (AI and KMW) extracted the data independently to standard data extraction forms piloted using three studies. Discrepancies were identified and resolved through discussion. For each study that passed initial screening, reviewed data was collected on the eligibility criteria (see Table 1) and an eligibility decision documented. In addition, data was sought and recorded for the variables in Table 2 for all included studies.

**Table 2:** Data collection variables for included studies

Type	Variable
Participants	Sample size (overall if single gate, control and target condition(s) if case-control) Study exclusion criteria Age and gender Other demographics
Methods and conditions	Site (country) Population (inclusion criteria, presentation, and demographics) Setting and number of centres Design Continuous sample? GI target condition(s) Index test: BSCA methodology or analytic technique, diagnostic criterion Level of technological development Threshold set prior? ROC used? Reference standard or independent diagnosis Flow and timing
Outcomes	Results of DTAs: AUCs, PPV, NPV, diagnostic odds ratio,sensitivity, specificity etc. Or results of other studies: statistical test for association/heterogeneity/correlation between index test and GI condition—*p* values
Other	Funding sources Conflict of interest

### Risk of bias in individual studies

The breadth of studies included made use of a standard quality assessment tool problematic. Hence, bias was assessed at the study level using a modified tool. The QUADAS-2 tool for Quality Assessment for Diagnostic Accuracy Studies was heavily modified (by KMW) with input from three validated checklists used in the quality assessment of a range of study designs for intervention studies: those of Downs and Black [10], Cho and Bero [11], and Moga et al. [12]. The tool was piloted and amended by two independent reviewers (AI and KMW). The resultant tool covered selection bias, performance bias, attrition bias, and reporting bias, as well as competing interests. Across the domains, some questions were retained or added that were applicable to all type of studies, e.g. domain 1: patient selection included ‘Did the study avoid inappropriate exclusions’ (from QUADAS-2) and ‘Were the characteristics of the cohorts clearly described’ (derived from Downs and Black [10]). Consideration of the checklists, especially Cho and Bero [11] and Moga et al. [12], prompted us to add additional questions on statistics and competing interests (see domains 5 and 6).

Elsewhere in the tool, pairs of signalling questions, one question for the DTA studies and one for the preliminary studies testing for associations or differences across groups (see Additional file 2), were used that each addressed the same aspect of bias. For example, the first signalling question for a DTA study was ‘Was a case-control (two-gate) design avoided?’ (derived from QUADAS 2), whilst for a preliminary study, it was replaced with ‘Were the control subjects/cohorts appropriate (similar population to those with the GI condition, matched or random)?’ (derived from Cho and Bero [11]).

Given the simpler preliminary or proof-of-concept studies tend to intrinsically have a higher rate of bias due to a case-control design, the question set used was noted and the results are presented separately below.

Some studies included two components: a test for an association and a subsequent ROC analysis. These were assessed twice, with the appropriate questions for the different parts of the studies.

Quality assessments of all included studies were performed by two independent reviewers (AI and KMW). In the event of disagreement after discussion to reach a consensus, a decision was made by a third reviewer (BJM).

## Results

### Study selection

**Fig. 1:**
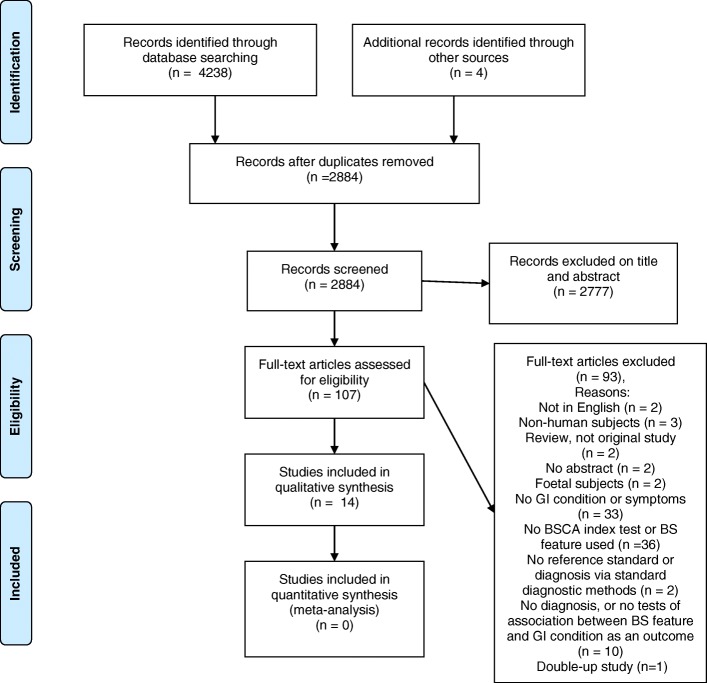
PRISMA flow diagram

We retrieved studies using the search protocol specified in our protocol. The database searches uncovered the following numbers of studies: Embase, 1421; IEEE, 288; Medline, 753; and PubMed, 1776. After discarding duplicates, 2880 studies remained. An additional four studies were uncovered by reading the references. One reviewer discarded 2770 papers after reviewing the titles and/or abstracts. One hundred and seven studies were excluded following assessment of the full text by two independent reviewers making consensus decisions. One paper was included after discussion with a third reviewer. Many studies retrieved from the medical databases searches were excluded due to a lack of computational analysis of bowel sounds. In parallel, many studies retrieved from the IEEE search were discarded due to a lack of clinical investigation, i.e. no GI diagnosis offered or no comparison with healthy controls. Several papers with the most sophisticated technology and pattern recognition analysis were excluded for simply outlining methods for identifying bowel sounds, or because the authors mimicked gastrointestinal conditions through the administration of drugs. Thus, ultimately only 14 studies were included in this review. The study selection process is detailed in Fig. 1.

### Study characteristics and results

A summary of the 14 included studies is given in Table 3. The studies cover a variety of target conditions, index tests, and study type.

**Table 3:** Characteristics and results of included studies

Paper	Target condition	Study design and population	Setting	Technology level	Technology (hardware and analysis)	Feature	Reference standard
Craine et al. 1999 [13]	IBS	Case-control using healthy controls (2 gate): healthy (15) and IBS (18) matched for gender bias. Heterogeneity test and ROC analysis on one dataset.	GI clinic in a county medical centre. USA	Low	Electronic stethoscope lower right quadrant. Analysis of sounds (150–450 Hz)	Six metrics and difference between fasting and fed states. Useful: fasting s-s interval (2 min recording)	Rome criteria for IBS. Symptoms > 6 months.
Craine et al. 2001 [14]	IBS and Crohn’s disease	Case-control using healthy controls and alternative diagnosis groups (3 gate): healthy (37), IBS (45), and Crohn’s (25). Crohn’s younger. ‘Healthy’ comprised community volunteers and patients attending GI clinic for diagnoses unrelated to abdominal discomfort. Heterogeneity test and ROC analysis on one dataset.	GI Clinic in a county medical centre. USA	Low	One electronic stethoscope to the right of umbilicus and ‘Enterotach’ analysis (start and stop times of sounds 150 to 450 Hz)	Fasting s-s interval (2 min recording)	Rome criteria. Clinical, radiological, and biopsy findings for Crohn’s disease.
Craine et al. 2002 [15]	IBS and non-ulcer dyspepsia (NUD)	Case-control using healthy controls and alternative diagnosis groups (3 gate): controls (10), IBS (11), and NUD (19) (split into 2 groups on bowel sound characteristic). Controls attending clinic for diagnoses unrelated to abdominal discomfort. Heterogeneity testing for multiple features.	GI Clinic in a county medical centre. USA	Moderate	Three electronic stethoscopes. Analysis enterotachogram (start and stop times of sounds and magnitude of sound envelope)	Sound mapping and measurement of freq and s-s interval.	Rome II criteria.
Hadjileontiadi*s* et al. 1999 [16]	IBS, diverticular disease (DD), bowel polyp (2 cm) and ulcerative colitis (UC)	Case-control (multi-gate): healthy (9), pre-confirmed diseases of the large bowel (7) (IBS, diverticular disease, 2 cm bowel polyp, ulcerative colitis. No details on incl. criteria given. Scatterplot analysis.	Unclear, probably hospital. Greece.	Moderate	Audioscope. Denoised with WTST-NST filter	Three procedures for analysis based on higher order crossings (HOC): scatter plots, distance from white Gaussian noise, calculation of the weighted φ2 statistic. 16 min BS recordings (8 min each) above right and left anterior superior iliac spine.	Unclear—“pre-confirmed”
Yoshino et al. 1990 [17]	Intestinal obstruction (large and small bowel)	Case-control with healthy controls (2 gate): healthy (4) and intestinal obstruction (21) (later split into 17 with simple obstructions and 4 with strangulating). Tests of association.	Hospital. Japan	Low	Foam covered microphone. Right lower abdomen.	Categorised sounds into three types based on frequency characteristics: Peak freq the most common freq). The upper and lower limits of freq range. Freq over 900 kHz present or absent (15 mins recording)	Obstructions were diagnosed based on clinical examinations including plain film based X-rays or laparotomy.
Ching et al. 2012 [18]	Small and large bowel obstruction	Cross-sectional (single -gate): 71 patients with suspected bowel obstruction (split into acute bowel obstruction, subacute bowel obstruction and no bowel obstruction). Little info on incl. criteria. Tests of heterogeneity across groups.	General hospital. Singapore	Low	Electronic stethoscope.	Sound duration, sound to sound interval, dominant frequency and peak frequency from 6 tracks of 8 s: 2 at each of 3 locations on lower abdomen.	Radiological imaging: plain film radiology in all, and CT in 85.9% of patients, and symptoms and physical signs.
Sugrue and Redfern 1994 [19]	Acute abdomen, varying severity (appendicitis, cholecystitis, and intestinal obstruction)	Case-control (multi- gate): healthy (63) and patients with an acute abdomen (61) (multiple conditions: appendicitis (25, 18 acute, 7 perforated), obstructions (21, 12 large, 9 small), cholecystitis (15))	Teaching hospital. Ireland	Low	Microphone (range 30–15,000 Hz) taped to right iliac fossa. 4 computer programs.	Ten-minute recordings (after fasting for 2 h in the case of controls). Five features: sound length, number of sounds, sound amplitude, silence length, sound/silence ratio.	Surgery and histology (for all but one)
Kim et al. 2011A [20]	Delayed gastric emptying	Case-control with healthy controls (2 gate): healthy (12), patients with spinal cord injury and delayed gastric emptying (4). Tests of heterogeneity across groups. Test of correlation between index and reference tests.	Unclear, assume University Hospital, Korea	High	Piezo-polymer sensor (range 8–2200 Hz) multiple filters. Denoising, segmentation, feature extraction (jitter and shimmer). R3 channel recordings (right upper quadrant, left upper, and left lower quadrant.	Nine features (jitter, shimmer, and trace) used to model eCTT. Fasting conditions, 200 g test meal at 9:00 am, then 10-min recordings at 9:30 am, 1:00 pm, and 5:00 pm.	Metcalf’s method. Ingestion of radiopaque marker and X-rays, to calculate total CTT.
Kim et al. 2011B [21]	Delayed gastric emptying	Case-control with healthy controls (2 gate): healthy (12), patients with spinal cord injury, and delayed gastric emptying (6). All male, controls younger. Tests of heterogeneity across groups. Used K-fold cross-validation to calculate correlation between index and reference tests.	Unclear, probably university hospital, Korea	Super high	Piezo-polymer sensor (range 8–2200 Hz) multiple filters. Denoising, segmentation, feature extraction (jitter and shimmer). Training and estimation of the back propogation neural network. 3 channel recordings (right upper quadrant, left upper, and left lower quadrant.	Six jitter and shimmer features used to model eCTT. Model refined through an artificial (back propogation) neural network. Fasting conditions, 200 g test meal at 9:00 am, then 10-min recordings at 9:30 am, 1:00 pm, and 5:00 pm.	Metcalf’s method. Ingestion of radiopaque marker and X-rays, to calculate total CTT.
Tomomasa et al. 1999 [22]	Pyloric stenosis and impaired gastric emptying in infants	Case-control with healthy controls (2 gate): healthy (6), infants with infantile hypertrophic pyloric stenosis (15). Similar ages. Heterogeneity study (SI across 2 groups HPS and healthy) and correlations between SI and standard measure of gastric emptying.	Unclear, probably children’s hospital, Japan	Low	Condenser microphone sound sensor attached with electrocardiograph tape 3 cm below umbilicus, for 60 min (when fasted) before pyloromyotomy, and at 9 to 12 h, 20 to 24 h, 40 to 48 h, and 112 to 120 h after the operation. Recordings made when sleeping (for at least 20 min.)	Sound index (SI) as the sum of absolute signal amplitudes expressed as volts per minute.	Gastric emptying measured using marker dilution-double sampling method. Diagnosis of pyloric stenosis (based on ?).
Spiegal et al. 2014 [23]	Post-operative ileus	Case-control (3 gate): healthy controls (8), patients with post-operative ileus (25), post-operative patients tolerating feeding (7). Controls 62.5% male, patients 100% male. Heterogeneity test across groups and an ROC analysis on differentiation between healthy and POI on the same dataset (note, those tolerating feeding not in ROC analysis)	1 teaching hospital. USA	Unclear (few details provided)	AGIS’ sensor with microelectronic microphone and computer to calculate motility scores.	Intestinal rate (average rate of pulses resulting from motility events), in a 60 min recording (post meal in controls).	Pragmatic definition of POI: presence of one or more of (1) nausea that precluded advancement of diet beyond sips on POD #1 or later, (2) post-op vomiting that precluded any oral intake, or (3) nasogastric tube decompression
Kaneshiro et al. 2016 [24]	Post-operative ileus	Cross-sectional (single gate) longitudinal prospective: subjects recovering from colorectal surgery (28). Consecutive sample. Identified an algorithm that maximised predictive discrimination. ROC analysis to assess sensitivity and specificity using the same data.	3 hospitals. USA	Unclear (few details provided)	AGIS’ sensor with microelectonuc microphone. 2 sensors either side of the umbilicus.	Intestinal rate (number of acoustic motilty events per minute. Metrics used were a drop in IR between POD1 and POD 2, plus % time that the subject had an IR below the 5th percentile.	Definition of POI was pragmatic. 3 criteria used.
Campbell et al. 1989 [25]	Diarrhoea—severe (post-gastrectomy) and mild idiopathic	Case-control (3 gate): healthy (22), severe diarrhoea (5), and mild diarrhoea (7). Ages differed between groups. Heterogeneity test and correlation between ref standard and index test values (additional test on effect of cisapride)	Unclear, probably teaching hospital. UK	Low	One transducer. Filter, integration, fast fourier transform, and ‘SVA’ analysis.	Post-prandial 3.5-h recording. SVA values expressed in linear energy units.	Oral caecal transfer time (hydrogen breath technique)
Liatsos et al. 2003 [26]	Small volume ascites	Case-control with healthy controls (2 gate): healthy (20), cirrhotic patients (with proven small-volume ascites) (20). Healthy slightly younger. Analysis of bowel sounds using a higher order crossings based technique to distinguish between the two groups.	Hepatobiliary and Liver Transplantation Unit, teaching hospital. UK	Moderate	Electronic stethoscope in a semi-soundproofed room. Right upper and lower abdomen. Subject lying supine.	16 min per patients. Fasted. WTST-NST Filter, denoised bowel sounds, HOC analysis, linear discrimination classification.	Small volume ascites picked up on ultrasound, but not clinical examination.

The majority of studies were preliminary in nature, i.e. case control (multi-gate or two-gate studies) to test for an association between a bowel sound feature or features and a target condition. Thus, they typically examined the potential usefulness of bowel sound analysis in diagnosis rather than true measures of diagnostic accuracy. Four studies involved ROC analysis to determine test cut-off points and provided data on sensitivity and specificity. In three cases, this was derived from multi-gate data [13, 14, 23]. The fourth study had a prospective, blinded, cross-sectional (single-gate) design, undertaken at multiple centres, and we would expect that this gives a better indication of accuracy in the clinical setting in which the test will be applied [24].

In two studies, the diagnostic outcome was continuous rather than discrete [20, 21]. Here, the authors assessed the degree of correlation between a continuous variable (colon transit time) calculated by standard methods and values estimated from models derived from bowel sound features.

These two studies employed relatively sophisticated acoustic signal processing and modelling techniques. However, typically most studies involved only rudimentary bowel sound analysis techniques, and this is reflected in the simplicity of the analytical techniques employed. The duration of bowel sound recordings used was generally short, but ranged from 16 to 1 h. In addition, in most studies, the level of computational analysis was relatively low: simple signal processing rather than advanced pattern recognition. In only one case was there an attempt at localization of the bowel sounds.

### Risk of bias and concerns about applicability within studies

**Fig. 2:**
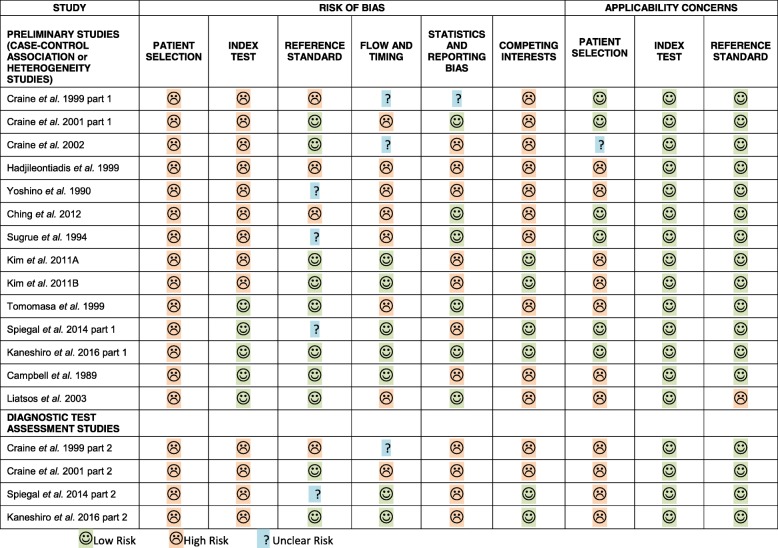
Outcomes of modified QUADAS-2 (quality assessment for diagnostic accuracy studies) assessment for included studies. The modified tool included two sets of signalling questions, one for the DTA studies and one for the preliminary studies testing for associations or differences across groups. The results of the two types of study are presented separately

Consensus decisions between two reviewers were reached for all sub-sections of the modified QUADAS tool for all articles included. The majority of studies were preliminary studies with case-control design, which typically are poorer in quality due to limited challenge bias and spectrum effects [27, 28]. These are grouped separately in Fig. 2. Generally, there was a high risk of bias in both the included DTA and preliminary studies.

Domain 1, patient selection, frequently gave rise to a high risk of bias because of a lack of information regarding study participant characteristics and inclusion/exclusion criteria. Three of the DTA studies had a case-control design. In addition, sample sizes were typically small which may have negatively impacted the reliability of results. For example, Kaneshiro et al.’s study [24] was well designed (blinded, prospective, longitudinal, single-gate (cohort) study across multiple centres, featuring a consecutive sample of patients), but it had only a small sample size of 28 participants, only nine of whom developed post-operative ileus.

There were no concerns about the applicability of the index test in any of the studies. However, we determined that it could have given rise to bias in over half of the preliminary studies and all the DTA studies. In all these, the bowel sound analysis was objective or, less frequently, was conducted blind [24] or prior [26]. However, in all cases, we determined that the index test could still have given rise to bias because the bowel sound feature or threshold was not pre-specified [13–16, 18, 19, 21, 23, 24]. Tests of association or correlation between the target condition and a range of different features were made to find one of interest, or the cut-off threshold was determined as part of the study.

Domain 3, the reference standard, was considered to lead to a high risk of bias in three preliminary studies. In Craine et al.’s 1999 IBS focused paper [13], there was a lack of clarity on two counts, and ratings of unclears led to us reporting a high risk under our protocol. The reference standard was the Rome II criteria which lacks reliability in the absence of other investigations. It was also unclear whether the diagnosis was made without knowledge of the bowel sounds analysis results. Similarly, we rated Hadjileontiadis et al.’s [16] study as having a high risk of bias in this domain since it was unclear on reference standard reliability and timing relative to bowel sounds analysis. Ching and Tan’s [18] study on bowel obstruction was considered susceptible to a high level of bias because the reference standard was not objective and was made after the bowel sound analysis (diagnosis confirmed by clinical follow-up, by clinical evaluation, and by radiological and operative findings). There was an unclear risk of bias in the further three studies, where we could not determine if the diagnosis was made without knowledge of the bowel sound analysis or not [17, 19, 23]. This problem was rectified in the second study with the AGIS system [24] where there were different teams undertaking clinical assessment and bowel sound analysis.

In all but one case, there was no cause for concern about the applicability of the reference standard to the review question. There was concern that the target condition in the study by Liatsos et al. [26] small volume ascites as defined by the reference standard (without knowledge of patient comorbidities) is not solely a GI condition. Indeed, this was the one paper where the decision on inclusion in the review had to be made by the third reviewer (BJM).

Flow and timing were poorly described in two studies by Craine et al. of bowel sounds analysis in relation to IBS and other conditions [13, 15], leading to an unclear risk of bias. In a third study by the same group [14], not only was the interval between index test and reference standard test unclear, but it also appears that a mix of different methods was used to diagnose Crohn’s disease. Similarly, in four other studies [17–19, 26], there was a variation in the reference standard used for subjects. In Tomomasa’s study [22], perhaps for ethical reasons, the healthy infant controls did not undergo a standard test to assess the rate of gastric emptying, and this introduced a risk of bias to the study.

Not all data was included in the analysis for two studies. In Ching and Tan’s study [18] of intestinal obstruction patients, six recordings were of poor quality and so were not included in the analysis, whilst the results from an IBS patient were not mentioned in the results section of the Hadjileontiadis et al. paper, which was also unclear on the relative timing of the reference standard and index tests.

The overall quality of statistics and reporting was disappointing for several studies. Problems related to statistics were found for two of Craine et al.’s studies [13, 15]. We determined that some of the statistics used in their 2002 study [15] were not appropriate. They looked for heterogeneity in sound to sound interval between groups, but since the non-ulcer dyspepsia groups were split based on this, the approach was circular. We were unable to determine if some of the statistics reported in an earlier Craine et al. paper [13] were appropriate since the test used was not detailed leading us to give them an unclear risk of bias in this domain.

The paper by Hadjileontiadis et al. [16] had quite sophisticated analysis but was still deemed to be at risk of bias due to statistics or reporting bias. No *p* values were provided, simply scatter plots and the IBS data was missing.

Four other studies had missing test outcomes [17, 21, 25]. Exact *p* values were missing in the two papers on delayed gastric emptying [20, 21], and one on acute abdomen [19].

The statistics on heterogeneity across groups for the first component of the first paper on the AGIS system [23] were considered to add bias, since the small sample sizes meant that non-parametric tests would have been more appropriate. This was rectified in their second paper, which detailed a similar method of bowel sound analysis for post-operative ileus diagnosis [24].

The DTA studies were all considered to have a high risk of statistical bias since the statistics provided on measures of accuracy were all derived from the same data from which the cut-off thresholds were determined, rather than from independent cases.

The pattern observed in risk of bias in relation to competing interests largely reflects the date of publication of the studies. Absence of information about competing interests and/or funding led to us record high risk in this domain for the earlier publications. However, we believe this was largely because many journals have only recently required statements in these areas.

## Results of individual studies

The results of all included studies are included in Table 4.

**Table 4:** Results of individual studies

Paper	Target condition	Main findings
Craine et al. 1999 [13]	Irritable bowel syndrome (IBS)	Fasting s-s interval useful: significant difference between IBS and healthy individuals ((*t* test) *p* < 0.0001).Using 640 msec as the cut-off, sensitivity was 89% and specificity was 100% on the preliminary data (AUC = 0.99)
Craine et al. 2001 [14]	IBS and Crohn’s Disease	Useful: fasting s-s interval is higher in Crohn’s and healthy individuals than in IBS individuals (heterogeneity across 3 groups (Kruskal Wallis) *p* < 0.0001). Using an s-s interval of 740 msec gave a sensitivity (NPV) of 97.8%, and TPV of 13.5% for distinguishing between IBS and controls (AUC = 0.978). The AUC for distinguishing CD from IBS patients was 0.843. High s-s interval in an individual with IBS symptom should prompt a search for an alternative diagnosis such as Crohn’s. Unable to differentiate between healthy and Crohn’s individuals based on this feature. The AUC for distinguishing CD from controls was only 0.709.
Craine et al. 2002 [15]	IBS and non-ulcer dyspepsia (NUD)	Useful: significant differences across all groups in s-s interval (Kruskal Wallis) *p* < 0.0001. Control vs IBS significantly different in % power in lower freq sounds, especially in right lower quadrant (RLQ) (*p* < 0.001). Also, significant differences between NUD and controls in ratio of gastric sounds to RLQ sounds (*p* < 0.001). Fewer differences between FGID groups, but IBS and NUD patients significant differences in ratio of gastric to RLQ sounds (*p* < 0.001). Note, the authors split the NUD patients into two groups based on s-s interval.
Hadjileontiadis et al. 1999 [16]	IBS, diverticular disease (DD), bowel polyp (2 cm) and ulcerative colitis (UC)	Useful: limited statistics, but scatter plots of HOC using the optimum HOC domain discriminate between patients and controls. The φ2 (non-weighted) statistic or with weights adapted to the HOC with maximum discriminative information, provides another simple discriminative feature between controls and DD and between DD and UC.
Yoshino et al. 1990 [17]	Intestinal obstruction (large and small bowel)	Useful: objective indicator of surgery for intestinal obstruction. Seriousness could be identified from bowel sounds characteristics—objective measure, and suggests treatment regimen—conservative or operative. Seriousness order: sounds type3 > sound type 2 > sound type 1.Those with type three sounds all had strangulating obstructions or a condition requiring surgery. Fewer of those with type 2 sounds required surgery (after a longer delay than group3 cases) and all of those with type 1 sounds were simple obstructions which did not require surgery. Upper and range of sound frequencies were higher significantly higher in type 1 than normal (*p* < 001). Peak (*p* < 0.001) and upper (*p* < 0.01) frequencies were higher in type 2 relative to type 1. Peak, upper, and range was significantly higher in type 2 relative to normal (*p* < 0.001).Type 3 significantly different from normal in peak (*p* < 0.001) and upper (*p* < 0.001) freq. Type 3 is significantly different from type1 in peak freq (*p* < 0.01), but there were no significant differences between type 1 and type 2 in sound frequencies.
Ching et al. 2012 [18]	Small and large bowel obstruction	Non-specific for diagnosing bowel obstruction. No sig diffs between the 3 groups (no obstruction, subacute, acute) in sound to sound interval, sound duration, dominant freq, and peak freq when look at all cases. However, incidence of prolonged bowel sounds increased significantly across the 3 groups in the suspected large bowel cases (*p* = 0.025). The bowel sounds may be useful in locating the site of an acute obstruction. Sound duration (*p* = 0.021) and the dominant frequency (*p* = 0.003) were significantly higher in large bowel obstruction vs small bowel obstruction. No bowel sound feature correlated with bowel calibre. Some indication of severity: sound to sound interval longer in the small bowel obstruction group that underwent surgery (*p* < 0.01).
Sugrue and Redfern 1994 [19]	Acute abdomen, varying severity (appendicitis, cholecystitis and intestinal obstruction)	Useful: mean number of bowel sounds was greater in normal subjects than those with appendicitis (*p* < 0.05) and obstruction (*p* < 0.05). Bowel sounds not significantly different in length for appendicitis and controls. However, sound to silence ratio was less in appendicitis (more silence) than in controls (0.05). Sounds significantly longer in cholecystitis and intestinal obstruction than in controls and those with appendicitis (*p* < 0.05).
Kim et al. 2011A [20]	Delayed gastric emptying	Useful: this method could be used for the non-invasive measurement of bowel motility. Jitter and shimmer of the bowel sounds of healthy group members were higher than those with spinal cord injury. Correlation coefficient between CTTs and eCTT was 0.987 (S.E. = 7.99 h)
Kim et al. 2011B [21]	Delayed gastric emptying	Useful: bowel sound features could be clinically useful for measurement of bowel motility. Jitter and shimmers of normal subjects were significantly higher than patients (*p* < 0.01). Performance of the algorithm: 12 random feature datasets used to train the model and 6 datasets used to test the algorithm. Outcome: correlation coefficient between CTT and eCTT was 0.89 (mean average error = 10.6 h). Estimation errors slightly better than the regression model derived from this data (similar to that used in Kim et al. 2011A).
Tomomasa et al. 1999 [22]	Pyloric stenosis and impaired gastric emptying in infants	Useful: decreased gastrointestinal sounds are suggestive of HPS and a useful indicator of gastric emptying and bowel motility after pylormyotomy. Mean SI was significantly less in pyloric stenosis patients before surgery than in healthy controls (*p* = 0.0013). Incidence of post-op symptoms negatively correlated significantly with SI at 24 h post-op (*p* = 0.035, R^2^ = 0.373). There was a significant positive correlation between SI and gastric emptying (*p* = 0.018).
Spiegel et al. 2014 [23]	Post-operative ileus	Useful: there is a relationship between intestinal rate and post-op clinical status. Significant differences between the three groups. However, there is some overlap between POI and re-feeding group, so only indicative. ROC analysis on differentiation of healthy controls (not the re-feeding group) and the POI group revealed a threshold of 0.1 events per second to give an AUC of 0.995.
Kaneshiro et al. 2016 [24]	Post-operative ileus	Useful: for the 5 day post-op period, intestinal rate (IR) was significantly lower in the POI group. Drop in IR between POD 1 and 2 observed in the POI group was sign diff from the increase seen in the non-POI group. % time IR was below the 5th percentile, also differed significantly. Used these last two variables to predict POI. ROC area under the curve was 0.83. Using a test threshold of 0.4, able to differentiate between groups with sensitivity 63%, specificity 72% and NPV 83%. High NPV suggests use of negative test result as a rule-out-tool for POI to aid decision making around diet advancement.
Campbell et al. 1989 [25]	Diarrhoea—severe (post-gastrectomy) and mild idiopathic	Limited usefulness: SVA significantly greater in the severe diarrhoea group than the healthy controls (*p* < 0.01). Difference not significant between mild and severe and mild and controls. Inverse correlation between SVA energy value and OCTT *p* < 0.01 (Spearman’s rho = − 0.486). Drug stimulation of the GI tract caused a significant increase in SVA measurements.
Liatsos et al. 2003 [26]	Small volume ascites	Useful: novel diagnostic features of bowel sounds identified that could give rise to a new diagnostic tool in routine clinical practice. There was a distinct separation of all cirrhotic patients with small ascites from controls (*p* < 0.0001). Coincided with radiological findings.

### Synthesis of results

The heterogeneity in index tests and target conditions precluded statistical meta-analysis. However, we are able to present a narrative describing the study results organised by target condition and bowel sound analysis approach.

#### Irritable bowel syndrome

Three studies [13–15] from one research group were included with a strong focus on IBS. The index test and technology (Enterotach analysis system) were similar in all three. The first of the studies [13] revealed that the mean sound-to-sound (s-s) interval from a 2-min recording was significantly shorter for the IBS group than healthy controls. The study included a ROC analysis with good results (AUC was 0.99 (*p* = 0.0001)). Similar highly significant results were found in the second study [14] with a slightly different cut-off value for the median s-s interval and also in the third study. The third study [15] also indicated that the percentage power in lower frequency sounds also differed significantly between IBS patients and healthy controls. The group were only able to differentiate between IBS and Crohn’s with much lower accuracy [14].

#### Inflammatory bowel disease

As mentioned, Craine et al. found that the s-s interval was higher in Crohn’s individuals than healthy subjects, but they were unable to reliably differentiate between the two based on this feature [14]. They still concluded that their technology could be useful in differential diagnosis, since high s-s interval in an individual with IBS symptoms should prompt a search for an alternative diagnosis to IBS, such as Crohn’s.

Hadjileontiadis et al. [16] processed bowel sounds based on higher-order crossings. Their statistics were limited, but scatter plots allowed discrimination between patients with ulcerative colitis, diverticular disease, a large bowel polyp, and healthy controls.

#### Intestinal obstruction

Yoshino et al. [17] used spectral analysis of bowel sounds to categorise sounds into three groups based on frequency characteristics. The groups corresponded to the severity of intestinal obstruction.

Ching and Tan [18] found mixed results. Their study examined four bowel sound features from six short recordings each made from patients with suspected bowel obstruction. The features were non-specific for diagnosing bowel obstruction. There were no significant differences when they looked at all cases. However, when they examined the large bowel obstruction cases alone, incidence of prolonged bowel sounds increased significantly from 4% with no obstruction to 11% with subacute obstruction, and 17% with acute obstruction. The authors also found that one feature provided indication of severity in small bowel obstruction cases. They also concluded that bowel sounds may be useful in locating the obstruction site; two features varied significantly between large and small bowel obstruction cases.

Intestinal obstruction was one of a range of causes of acute abdomen considered by Sugrue and Redfern [19]. Different sound features allowed differentiation between the different causes, although their findings suggest that analysis of multiple features would be needed for full differential diagnosis. They found that the mean number of bowel sounds was significantly less in subjects with obstruction and appendicitis than in normal subjects. In addition, sounds were significantly longer in cholecystitis and intestinal obstruction than in controls and those with appendicitis.

#### Appendicitis

Sugrue and Redfern [19] also found that the fasting sound to silence ratio was less in appendicitis cases than in controls, although bowel sounds were not significantly different in length.

#### Delayed gastric emptying and reduced bowel motility

More sophisticated processing and modelling techniques have proven useful in measurement of bowel motility and characterisation of delayed gastric emptying. Two studies were undertaken by Kim et al. [20, 21] on largely the same data set, with extraction of jitter and shimmer features from bowel sounds recorded from three channels. The subjects were male patients with spinal cord injury and delayed gastric emptying and healthy controls. Both the jitters and shimmers of the normal subjects were higher than those of the patients, and their colon transit times (CTT) were lower (*p* < 0.01). The first study [20] employed regression modelling based on stepwise selection methods to select the optimal nine features with which to model CTT. The correlation coefficient, coefficient of determination (*R*^2^), standard error (SE), and the absolute differences between the CTTs and eCTTs were 0.987, 0.974, 7.99, and 3.5 ± 3.3 h. The average absolute error on the cross-validation test was 7.3 ± 2.4 h. The same team undertook quantitative estimation of the CTT using an artificial neural network model of acoustic features, on the same data plus two additional patients, with slightly improved results [21]. They obtained 18 jitter and shimmer features of colonic sounds. The top six features (correlation coefficient with measured CTT was 0.65 or above) were used for the input vector for the artificial neural network (ANN). The ANN model gave correlation coefficient, MAE, and RMSE between the CTTs and eCTTs of 0.89, 10.6, and 14.6 h respectively. The ANN model had the same correlation coefficient but smaller error than a regression model derived from the expanded data.

The authors concluded that the algorithm had good potential as a tool for the continuous and non-invasive monitoring of bowel motility, instead of, complementary to conventional radiography.

#### Pyloric stenosis and impaired gastric emptying in infants

Tomomasa et al. [22] found analysis of bowel sounds potentially useful as an indicator of gastric emptying and bowel motility for paediatric patients. They observed decreased gastrointestinal sounds (using the sum of the amplitude of sound signals as the sound index) in infants with hypertrophic pyloric stenosis before surgery (4.6 ± 1.0 mV per minute) compared to healthy controls (31.7 ± 8.4 mV per minute). They also found a significant negative correlation between incidence of post-operative symptoms and the sound index at 24 h post-op, and a significant positive correlation between the sound index and gastric emptying.

#### Post-operative ileus

The most comprehensive analysis of a BSCA approach comes from two studies of the acoustic gastrointestinal surveillance biosensor (AGIS) system used to determine post-operative clinical status. Spiegel et al. [23] investigated the relationship between AGIS-derived ‘intestinal rate’ and the healthy fed state versus two post-operative states: post-operative ileus (POI) and toleration of feeding. It is unclear from their description whether the intestinal rate refers to propulsive events occurring during the active phase of the migrating motor complex or other events. However, the authors did find significant differences between the three groups in the index.

ROC analysis was conducted on the preliminary data to assess differentiation between healthy controls and the POI group. A threshold of 0.1 events per second gave an AUC of 0.995. ROC analysis of the more clinically useful differentiation between patients experiencing POI and toleration of refeeding was not undertaken. Motility rates were significantly higher in fed versus POI patients (*p* = 0.017). However, the fact that there was overlap in the motility rate between the POI and feeding group indicated that the index could possibly be only indicative of status.

The group’s second study of patients recovering from colorectal surgery had a blinded, single-gate, longitudinal, prospective, multi-centre design and was more enlightening as well as being well-designed to reduce bias [24]. Using ROC analysis, the authors identified an algorithm that maximised predictive discrimination between POI and non-POI groups. The algorithm encompassed two metrics derived from the intestinal rate. The authors emphasised the high negative predictive value (NPV) because this meant that the AGIS system could offer confidence to hospital staff that POI is unlikely and that diet advancement would be safe. They found that a test threshold of 0.4 provided an NPV of 83%, sensitivity of 63%, and specificity of 72%. The study was only an interim report on the first 28 subjects of a 100 subject clinical trial, but it appears that the AGIS system will prove useful in both prognosis and diagnosis in the post-operative setting.

#### Other conditions

Liatsos et al. [26] found that filtered and denoised bowel sounds subjected to higher order crossings (HOC) analysis could prove useful in diagnosis of small volume ascites. This is a similar approach to that used by Hadjileontiadis et al. [16] (see above). Scatter-plots of third-order zero crossings reflected distinct differences in the sound transmission path for cirrhotic patients with small ascites and healthy controls and allowed separation of the two (*p* < 0.0001). A single gate study is now needed to confirm these findings.

Campbell et al.’s [25] study of surface vibration analysis (SVA) included comparison of two groups of patients: one with severe (post-gastrectomy) diarrhoea and the other with mild idiopathic diarrhoea. The SVA values were also compared to oral caecal transfer time. The authors found evidence of limited usefulness for the approach: SVA values were significantly greater in the severe diarrhoea group than in the healthy controls, but the differences were not significant between other groups.

## Discussion

### Implications and usefulness

Gastrointestinal disorders are a common cause of morbidity worldwide. There is a need for non-invasive, simple, diagnostic tests to reduce the demand for gastroenterology referrals and for investigations such as endoscopy and imaging with their associated risks. Our aim was to assess the potential for BSCA to meet this need.

Our review had several strengths. We conducted a highly comprehensive search of both the medical and engineering literature, without limiting study design type or GI condition to gain a full understanding of the applicability of BSCA. To allow quality assessment of the breadth of studies uncovered, we developed a novel quality assessment tool with parallel question sets for DTA and other study types.

All 14 included articles assessed associations or correlations between bowel sound features and GI conditions, many with highly significant results. Four of the studies also incorporated preliminary studies of diagnostic accuracy. These DTA studies provide only a moderate level of evidence to support the idea that bowel sound analysis is currently useful as a tool in GI diagnosis. However, together all the studies provide excellent evidence that many GI conditions are characterised by specific bowel sound features. Hence, there is strong evidence of the potential for future use of BSCA in diagnosis of GI disease and disorders.

The main diagnostic benefits or strongest associations between a bowel sound feature and condition were demonstrated in papers examining a single GI condition (e.g. IBS [13], ascites [26]) or where there was estimation of a single variable such as colon transit time [21]. However, it is important to note that the vast majority of these studies were case-control in design which can lead to inflated accuracy measures (see below).

Interestingly, the data also seems clearest where the target condition is associated with motility, e.g. hypomotility in post-operative ileus [24], disordered motility in IBS [13], delayed gastric emptying in adults [21] and infants [22], or prediction of colon transit time.

Whilst clinical applicability increases with two gate studies utilising control patients with conditions causing similar symptoms, incorporating multiple pathologies and variables appeared to make BSCA less reliable, or at least more difficult. In these cases, it necessitated study of multiple features, or recordings from multiple sites. For example, multiple features are needed to differentiate between causes of acute abdomen [19] and results were mixed in studies of bowel obstruction [17, 18]. The latter is perhaps surprising since traditionally, auscultation has been used in diagnosis of bowel obstruction with an expectation that this condition results in distinctive higher pitched sounds with a tinkling quality (Talley and O'Connor [29]).

The clearest and most reliable evidence for the utility of BSCA comes from tests of the AGIS system in diagnosis of post-operative ileus. Sensitivity and specificity were not overly impressive, but there is promise of a high negative predictive value, which is key in the clinical setting [23, 24]. The NPV varies with the prevalence of the target condition. However, given this study was undertaken in the setting in which the test will be used, we can be hopeful about the applicability of the results. The latest published study [24] is based on a small sample size; the full results from the clinical trial are needed to have full confidence in its utility. However, it appears that the AGIS system will prove useful in both prognosis and diagnosis in the post-operative setting. Certainly, it is likely that the system will provide useful additional information for the clinician to use in decision-making, even if it is not used as a stand-alone test. This work is particularly exciting because it also demonstrates that monitoring is possible with long-term recordings and real-time feedback.

Evidence from more rudimentary DTA-type studies suggested that BSCA may be useful in the diagnosis of IBS and in the differentiation between functional gastrointestinal disorders [13, 15]. In particular, it appears that study of the s-s interval could be used in IBS diagnosis. However, BSCA with technology at the level of included studies is unlikely to remove the need for endoscopy and imaging, especially given BSCA alone was not effective in differentiation between IBS and IBD.

### Future potential

Several studies were excluded from our review for reasons including a lack of GI disease, non-English full text, or no abstract [30–53]. Although of less value than included papers, some excluded papers add information to help us assess the feasibility of the overall approach and are worthy of discussion.

Some provide information specifically about the clinical possibilities for BSCA. Three studies were excluded because only their abstracts were in English; however, they appear to add weight to the argument for utility of BSCA in diagnosis of post-operative ileus [31–33].

Two short letters were excluded from the review, due to lack of abstract, but have some relevance to understanding of the utility of Craine et al.’s technology in the diagnosis of IBS and IBD [34, 35]. Yuki et al. [34] using the Enterotach technology found no significant effect on s-s interval in response to administration of pro-kinetic drugs or a stimulant laxative to mimic bowel disease. The authors suggested that the short recording time may not be long enough to detect alterations in the bowel sounds. In response, Craine and Silva [35] suggested that the effects of drugs on bowel sounds may not be easily predicted. They suggested that the decreased s-s interval reflects disordered motility rather than increased motility and sited unpublished data showing that both diarrhoea-dominant and constipation-dominant IBS patients have markedly increased fasting rates of sound production. Yuki et al. [34] were also unable to differentiate between inflammatory bowel disease patients and normal controls using the Enterotach analysis system.

However, overall, there were very many excluded papers that support the idea that BSCA could be useful. Some studies included patients with GI disease but were excluded because there was no independent reference standard for diagnosis. For example, Yamaguchi et al. [36] looked at the bowel sounds of diabetic patients with delayed gastric emptying. They found a lower sound index for gastroduodenal sounds (sum of the amplitude) in diabetic patients after food intake when compared to controls. Similarly, Goto et al. [37] studied bowel sounds in patients with sepsis and found a feature that negatively correlated with interleukin-6 blood concentration. Ozawa et al. [38] found reduced bowel sounds in Parkinson’s disease and multiple system atrophy patients. These studies hint at the breadth of conditions BSCA could be applicable to in the future. Logic would also suggest that pathology affecting the gut lumen (e.g. luminal masses, stricturing disease, or diverticular disease) could also be diagnosed via BSCA, which could provide an alternative screening test for colorectal malignancy.Other studies excluded were those utilising drug administration as a mimic of GI disease. Tomomasa et al. [39] and Emoto et al. [30] delivered drugs known to affect bowel motility, e.g. cisapride, scopolamine, and mosapride, allowing the authors to document changes in bowel sounds in the post-prandial state. These studies prompt us to think it is possible that BSCA may also be useful in development of an objective monitoring tool to assess the effects of GI treatments or GI symptoms as patients undergo management. In addition, BSCA could be used as a tool to test for side effects on the GI tract of a whole range of medications.

Some excluded studies simply demonstrate that bowel sounds can be extracted from the wealth of sounds emanating from the abdomen and surrounding environment, processed, and analysed [40, 41]. These are vital first steps in the use of BSCA for diagnosis. Many of these papers were more recent and indicate advancement of technology and more sophisticated analysis, which may provide powerful results if ever applied to a clinical setting [40, 42–46]. They certainly throw into stark relief the very simple analysis on short recordings undertaken in the older studies, such those by Craine’s group [13].

We note that increased computer processing power has allowed use of longer recordings [40], which could be useful in the long-term monitoring of conditions, use of data from multiple sensors [46, 47], and real-time analysis [48]. The more recent studies also utilise signal processing techniques that are much more sophisticated [44, 45, 50–53]. In addition, we see exciting pattern recognition and machine learning techniques being employed [30, 46]. This approach is likely vital when needing to utilise large amounts of data, e.g. from longer recordings, multiple sensors, or multiple features.

### Limitations

Perhaps the most significant limitation on this review is the heterogeneity between studies preventing formal statistical analysis. For example, no two DTA studies employed exactly the same BSCA methodology with the same cut-off point being tested for a given target condition. Similarly, no two heterogeneity studies employed the same statistics to allow comparison.

Diversity of bias assessments for different articles warranted use of a heavily modified QUADAS tool. Whilst this adapted tool is not validated, it did include questions derived from QUADAS-2 and other checklists that have been validated. It would not have been possible to adequately assess these studies using a single existing validated tool because of the vast heterogeneity of included studies. Note, we still presented the results of the preliminary and DTA studies separately to highlight the general difference in quality.

We searched both the medical and engineering literature in order to undertake a comprehensive search but decided to only include peer-reviewed studies to ensure the reliability of studies. Hence, we did not search clinical trials registers. We gained advice on our search strategy but did not use the Peer Review of Electronic Search Strategies checklist and, due to limited human resources, only included studies in English.

Our screening strategy was limited in that only a single reviewer undertook initial screening, although in the event of uncertainty, a second person was involved.

A major limitation of the included studies themselves was that the majority were dated and only employed older techniques of analysis and recording of sound data. Other limitations of the included studies were revealed by the quality assessment process: small sample size, poor specification of reference standard used, and weak statistical analysis including preliminary tests for association. We are aware that we have recorded a high risk of bias for the majority of studies, which diminishes our confidence in the potential for BSCA usefulness. In part, this reflects the study design. Many were tests of association or case-control in design allowing comparison between two groups: healthy controls and individuals with well-developed disease status. Whilst this type of preliminary study is extremely useful to screen whether a diagnostic test is worth developing, it can lead to an overestimate of the likely sensitivity and specificity of the test when used in the clinical setting. Use of healthy controls leads to inflated estimates of specificity, since few will have other diagnoses that could generate false positives. Similarly, inclusion of individuals with advanced disease will generate fewer false negatives and hence increase estimates of sensitivity. It was refreshing to review the paper from Kaneshiro et al. with a more sophisticated single-gate (cohort) design and consecutive sampling.

However, in at least one domain (domain2: index test), we may have been too severe in our decision-making. Studies examining multiple features, rather than one pre-specified criterion, were assessed as being at high risk of bias [16, 20, 21]. Nevertheless, we are aware that in some cases, it was necessary to use multiple features to enable differential diagnoses through BSCA, and hence, there this proved a very effective approach.

Generally, there is a significant need for improved study design: including more cross-sectional (single-gate) DTA studies in the appropriate clinical setting. With time, multiple well-designed studies are necessary to allow for meta-analysis and greater confidence in our conclusions.

### Recommendations

Whilst hopeful about future prospects, we cannot yet recommend any BSCA-based diagnostic test to clinicians. The majority of included studies gave significant results, but only tested prototype technology and were simple preliminary case-control studies to test for association, or correlation studies. Where technology is closer to market and study design was more advanced [24], we still found problems in study quality, especially related to small sample sizes and patient selection. As mentioned above, replication of high-quality studies and larger sample sizes are necessary. Hopefully, this is likely to be addressed, for the AGIS system at least, in the next few years.

Generally, we found that researchers have much work to do to before BSCA can be applied in clinical practice. However, there is a great opportunity for gastroenterologists to work with engineers and software developers to provide access to patients in the correct setting and inform on good trial design. Together such collaborations should be able to provide clinically relevant high quality data, and possibly real progress in GI diagnostics.

## Conclusions

With increasing rates of endoscopy and CT scan requests, the potential for damage and associated risks is also increasing in gastroenterology patients. Computerised analysis of bowel sounds shows promise not only as a diagnostic tool but also as a tool for prognosis of GI disorders. There is a need for further DTA studies examining a standardised approach to recording bowel sounds for computerised analysis and proper statistical evaluation with the powers of modern technology. Similar advances in diagnostic tests of other areas of medicine are starting to reap benefits, although these largely involve image analysis rather than audio (e.g., in detection of retinal disease [54]). It appears that differential diagnosis is a more intractable problem and more complex approaches are needed for conditions not directly linked to motility. However, the associations recognised in studies included in this review reveal that BSCA may be a powerful tool in the diagnosis of a number of gastrointestinal conditions, once the technology is fully developed.

## Additional files

Additional file 1: Modified QUADAS-2 tool: risk of bias and applicability judgments. (DOC 96 kb)

Additional file 2: Completed PRISMA checklist. (DOC 63 kb)

## Abbreviations

AGIS: Acoustic gastrointestinal surveillance biosensor
ANN: Artificial neural network
AUC: Area under the curve
BSCA: Bowel sound computational analysis
CT: Computed tomography
CTT: Colon transit time
DD: Diverticular disease
eCTT: Estimated colon transit time
freq: Frequency
GI: Gastrointestinal
HOC: Higher order crossings
IBS: Irritable bowel syndrome
NPV: Negative predictive value
NUD: Non-ulcer dyspepsia
OCTT: Oral to caecal transit time
PICO: Population, index test, reference test, diagnosis
PIRD: Population, index test, comparator, outcome
POI: Post-operative ileus
PPV: Positive predictive value
PRISMA: Preferred Reporting Items for Systematic Reviews and Meta-Analyses
QUADAS: Quality Assessment of Diagnostic Accuracy Studies
RLQ: Right lower quadrant
ROC: Receiver operating characteristic
S.E.: Standard error
s-s: Sound-to-sound
SVA: Surface vibration analysis
UC: Ulcerative colitis
WTST-NST: Wavelet transform-based stationary–nonstationary
