# Supplementary material for: The potential of computerised analysis of bowel sounds for diagnosis of gastrointestinal conditions: a systematic review
Source: Syst Rev. 2018 Aug 17;7:124. doi: 10.1186/s13643-018-0789-3 (PMC6097214; doi:10.1186/s13643-018-0789-3)
Supplement: Supplementary file 1 — Modified QUADAS-2 tool: risk of bias and applicability judgments. (DOC 96 kb) [file 13643_2018_789_MOESM1_ESM.doc]

Modified QUADAS-2 tool: Risk of bias and applicability judgments

**Phase 1 Review Question**

**Overarching question: is Bowel Sound Computerized Analysis (BSCA) likely to be useful as a tool in GIT condition diagnosis?**

**Q 1. Can Bowel Sound Computerised Analysis be used to accurately diagnose specific gastrointestinal conditions?**

Patients with symptoms of GI conditions (specific to the target condition for each study). There may be multiple conditions in a single study.

Index test- a Bowel Sound Analysis criterion (varies across studies)

Reference standard: diagnosis by a clinician (specific to the target condition)

Target condition: A GI condition (varies across studies)

Where in the diagnostic pathway/setting also varies with the different conditions in terms of intended use of the index-test, patient presentation and prior testing.

The setting should be appropriate to the target condition, e.g., a clinical setting for a serious acute condition, but could be in the home, pharmacy or clinical setting for chronic functional gut disorder.

Intended use of the index test: diagnostic

Patient presentation: symptoms of the target condition (DTA studies)

Prior testing: prior testing restricted to clinical history and examination for DTAs

**Q.2 Does Bowel Sound Computerised Analysis identify signature patterns or criteria associated with GI conditions?**

Participants: patients with various conditions (physical, infection, functional) and healthy controls (varies with study, some may just have multiple target conditions).

Index test: bowel sounds recording and computerised analysis

Comparator/Control: Healthy controls, or different target condition groups

Outcome: statistical test result and characterisation of a sound ‘pattern’ associated with the GIT condition

Where in the diagnostic pathway/setting also varies with the different conditions in terms of intended use of the index-test, patient presentation and prior testing.

The setting should be appropriate to the target condition, e.g., a clinical setting for a serious acute condition, but could be in the home, pharmacy or clinical setting for chronic functional gut disorder.

Intended use of the index test: diagnostic

Patient presentation: Already has a diagnosis (Correlational studies).

Prior testing: for correlation studies it may include the reference test, and other tests.

Some questions are just for **DTA** studies, some for **Preliminary** = **non-DTA** (other types of studies) and some are for **All** studies.

**Phase 2: Review specific tailoring**

**Bowel Sounds Analysis and Diagnosis of Gastrointestinal Diseases and Disorders: A Systematic Review**

**Record authors and title.**

**Record if reviewing as a DTA or correlation/association study.**

| **Domain 1: Patient selection** | |
| --- | --- |
| 1. **Risk of bias (Selection Bias)** |  |
| **Describe methods of patient selection:**  (Record data upon which signalling questions will be answered. Provides transparency.) | |
| - **DTA study : Was a case-control (two-gate) design avoided?** | Yes/No/Unclear |
| - + **Non-DTA study: Were the control subjects/cohorts appropriate (similar population to those with the GI condition, matched or random)?** | Yes/No/Unclear |
| - **All: Did the study avoid inappropriate exclusions?** | Yes/No/Unclear |
| - **All: Was a consecutive or random sample of patients enrolled?** | Yes/No/Unclear |
| - **+ All: Was there an appropriate sample size?** | Yes/No/Unclear |
| - **+ All: were the characteristics of the cohort(s) clearly described** | Yes/No/Unclear |
| **Could the selection of patients have introduced bias?** | RISK: LOW/HIGH/UNCLEAR |
| 1. **Concerns regarding applicability** |  |
| **Describe included patients (prior testing, presentation, intended use of index test and setting):** | |
| **Is there concern that the included patients do not match the review question?**  **All:**  **+Was the design of the study appropriate to answer the study question?**  **+Did the study include patients (if one-gate design), or a group of patients (if case-control design), with the GI condition/symptoms the test will be used for?**  **+Was it a representative sample of those the test will be used for?**  **+Were the patients at a time-point in their diagnostic pathway representative of when the test would be used?**  **+Was the setting appropriate?** | CONCERN: LOW/HIGH/UNCLEAR |
| **Domain 2: Index test(s) *(if more than 1 index test was used, please complete for each test)*** | |
| 1. **Risk of bias** (Performance bias/blinding) |  |
| **Describe the index test and how it was conducted and interpreted:**  (Record data upon which signalling questions will be answered. Provides transparency.) | |
| - **DTA: Was the index test an objective test, and/or were the results interpreted without knowledge of the results of the reference standard?** | Yes/No/Unclear |
| - **+ DTA study: If a threshold or other ABS citerion was used was it pre-specified?** | Yes/No/Unclear |
| - **+ Other study: Was there a test for an association between the target condition and a pre-specified threshold/criterion? (threshold or just one parameter tested)** | Yes/No/Unclear |
| - **+ Other study: Was the bowel sound analysis undertaken for each particpant objective, or made without knowledge of the results of standard diagnostic testing or without knowledge of which groups the participant was in?** | Yes/No/Unclear |
| **Could the conduct or interpretation of the index test have introduced bias?** | RISK: LOW/HIGH/UNCLEAR |
| 1. **Concerns regarding applicability** |  |
| **Is there concern that the index test, its conduct, or interpretation differ from the review question? i.e.,**  **+ DTA study: Was there clear analysis of diagnostic accuracy for an ABS criterion with a condition?**  **Or**  **Was there clear analysis of diagnostic accuracy to differentiate between multiple conditions?**  **+ Other study: Was there analysis of heterogenity in ABS criterion values between healthy individuals and those with the target condition or between individulas with different target conditions?**  **Or**  **and/or analysis of association between ABS criteria (or a ABS criterion) and GI condition(s)?** | CONCERN: LOW/HIGH/UNCLEAR |
| **Domain 3: Reference standard** | |
| 1. **Risk of bias** (Performance bias/blinding) |  |
| **Describe the reference standard and how it was conducted and interpreted:**  (Record data upon which signalling questions will be answered. Provides transparency.) | |
| - **DTA: Is the reference standard likely to correctly classify the target GI condition?** | Yes/No/Unclear |
| - **DTA study: Was the reference test an objective test and/or were the reference standard results interpreted without knowledge of the results of the index test?** | Yes/No/Unclear |
| - **+ Other study: were the target, and if appropriate, the healthy conditions diagnosed via standard methods likely to correctly classify the target GI condition?** | Yes/No/Unclear |
| - **+ Other study: was the reference test an objective test or were the target, and if appropriate, the healthy conditions diagnosed without knowledge of the results of the bowel sound analysis?** | Yes/No/Unclear |
| **Could the reference standard, its conduct, or its interpretation have introduced bias?** | RISK: LOW/HIGH/UNCLEAR |
| 1. **Concerns regarding applicability** |  |
| **Is there concern that the target condition as defined by the reference standard does not match the review question? i.e.,**  **+ Is there a concern that the target condition as defined by the reference standard is not a GI condition?** | CONCERN: LOW/HIGH/UNCLEAR |
| **Domain 4: Flow and timing** | |
| 1. **Risk of bias** (Includes Attrition bias) |  |
| **Describe any patients who did not receive the index test(s) and/or reference standard or who were excluded from the 2x2 table (refer to flow diagram):**  **Describe the time interval and any interventions between index test(s) and standard diagnosis:** | |
| - **DTA study: Was there an appropriate interval between index test(s) and reference standard?** | Yes/No/Unclear |
| - **DTA study: Did all patients receive a reference standard?** | Yes/No/Unclear |
| - **DTA study: Did patients receive the same reference standard?** | Yes/No/Unclear |
| - **+Other study: Was there an appropriate interval between diagnosis (or reference standard test) and the bowel sound study?** | Yes/No/Unclear |
| - **+Other study: Did all patients receive a diagnosis via a standard method (or reference standard test)?** | Yes/No/Unclear |
| - **+Other study: Was the condition diagnosed by the same standard method in all cases, or was the reference standard the same in all cases?** | Yes/No/Unclear |
| - **All: Were all patients tested included in the analysis?** | Yes/No/Unclear |
|  |  |
| **Could the patient flow have introduced bias?** | RISK: LOW/HIGH/UNCLEAR |
| **Domain 5: Statistics and Reporting bias** |  |
| **A. Risk of bias** |  |
| **+Describe the statistical analysis approach used** | |
| - **All: +Were the statistical tests used to assess the main outcomes appropriate?** | Yes/No/Unclear |
| - **All: +Were exact p-values provided** | Yes/No/Unclear |
| - **All: +Were the results of all “outcomes” reported: index tests, tests of association etc.** | Yes/No/Unclear |
|  |  |
| **+ Could the statistics and reporting have introduced bias** | RISK: LOW/HIGH/UNCLEAR |
| **Domain 6: Competing interests** |  |
| **A. risk of bias** |  |
| **+ Describe the competing interests and souces of support for the study.** | |
| - **All: +Are competing interests reported?** | Yes/No/Unclear |
| - **All: + Are sources of support for the study reported?** | Yes/No/Unclear |
|  |  |
| **+ Could competing interests have introduced bias?** | RISK: LOW/HIGH/UNCLEAR |

**Guidance for Judgments**

**BIAS**

For each domain:

All yes leads to a LOW risk of bias.

1 no in the bias section leads to consideration of a HIGH risk of bias

2 no’s in the bias section leads to a HIGH risk of bias

One unclear, and zero NOs leads to an UNCLEAR risk of bias

Two unclears leads to high risk

**APPLICABILITY**

Domain 1, 2 & 3:

All yes leads to LOW concern

1 no in the applicability section flags consideration of a HIGH concern

2 no’s in the applicability section leads to HIGH concern

One unclear leads to a an UNCLEAR level of concern

Two unclears leads to high risk
